# Metal-Coordinated Polymer–Inorganic Hybrids: Synthesis, Properties, and Application

**DOI:** 10.3390/polym17020136

**Published:** 2025-01-08

**Authors:** Shaghayegh Abtahi, Nayanathara Hendeniya, Sharif Tasnim Mahmud, Gabriel Mogbojuri, Chizoba Livina Iheme, Boyce Chang

**Affiliations:** Department of Materials Science and Engineering, Iowa State University, Ames, IA 50011, USA

**Keywords:** Polymer-Inorganic Hybrids, coordination bonding, Metal-Coordinated Polymers, block copolymers, Synthesis Approaches, metallosupramolecular polymers

## Abstract

This review examines the recent advancements and unique properties of polymer–inorganic hybrid materials formed through coordination bonding (Class II hybrids), which enable enhanced functionality and stability across various applications. Here, we categorize these materials based on properties gained through complexation, focusing on electrical conductivity, thermal stability, photophysical characteristics, catalytic activity, and nanoscale self-assembly. Two major synthetic approaches to making these hybrids include homogeneous and heterogeneous methods, each with distinct tradeoffs: Homogeneous synthesis is straightforward but requires favorable mixing between inorganic and polymer species, which are predominantly water-soluble complexes. In contrast, heterogeneous methods are post-processing techniques that provide high area selectivity for inorganic precursors, allowing precise integration within polymer matrices. Finally, we highlight the role of hybrid linkers, namely metallosupramolecular polymers, in creating structural diversity. These can be organized into three main groups: metal–organic frameworks (MOFs), coordination polymers (CPs), and supramolecular coordination complexes (SCCs). Each of these groups introduces unique structural and functional properties that expand the potential applications of hybrid materials.

## 1. Introduction

Organic–inorganic hybrid materials (OIHs) have gained increasing attention due to their ability to combine the diverse properties of organic materials with the unique characteristics of inorganic components. Over the past two decades, these materials have been the focus of extensive research exploring a wide range of applications, including coatings, catalysis, optics, optoelectronics, and biomedical fields. Polymer-based hybrids are particularly desired due to the excellent processability of polymers [1,2,3].

The properties of OIHs are strongly influenced by the types and nature of the organic and inorganic precursors used in their synthesis. Inorganic precursors, such as metal salts or metal oxides, interact with organic polymers through functional groups like carboxylates, sulfur groups, and amines [4]. These inorganic components not only provide structural reinforcement but also introduce specialized functionalities, such as catalytic activity, luminescence, or conductivity, while enhancing the mechanical and thermal stability of the material. For instance, Zr(IV) and Ti(IV) ions create highly stable frameworks through strong coordination with organic linkers, making them well suited for harsh environments or high-temperature applications [5]. Additionally, the interaction between inorganic nanostructures and polymer matrices can enhance ionic conductivity by forming transport channels and amorphous regions that facilitate ion dissociation [6,7]. The coordination geometry and oxidation state of the metal ions also play a critical role in determining the structure and properties of the hybrid material. For example, Cu(II) ions are known to form square planar complexes that contribute to redox-active frameworks, while Fe(II) and Fe(III) ions provide tunable redox activity, leading to materials with unique magnetic and electronic properties [8]. Furthermore, the ionic radius of the metal ion affects the porosity of the hybrid material. Lanthanide-based hybrids, characterized by larger ionic radii, exhibit open architectures and greater spatial flexibility, making them more porous than those formed with transition metals like Zn(II) or Cu(II) [9,10].

Inorganic–organic hybrid materials can be categorized based on the dominant phase, either as organic–inorganic or inorganic–organic materials. However, this classification is not universally accepted due to the large number of intermediate cases. Unlike general nanocomposites, hybrid materials are distinguishable by the presence of chemical reactions between the organic and inorganic phases rather than being simple physical mixtures. Consequently, hybrid materials are typically classified according to the type of interaction occurring between the organic and inorganic components [3,11], namely Class I and Class II hybrids. Class I hybrids are characterized by weak interactions, such as Van der Waals forces, electrostatic interactions, or hydrogen bonds between organic and inorganic components. These hybrids often have a core–shell structure, where inorganic nanostructures are embedded in an organic matrix, making them suitable for applications like biosensors [12], photocatalysis [13], and optoelectronics [14]. However, to prevent phase separation, compatible functional groups on both the organic and inorganic components are essential [15]. In contrast, Class II hybrids feature strong chemical bonds, including covalent, ionic–covalent, or coordination bonds, between the organic and inorganic phases. These hybrids can include metal–carbon bonds and alkoxy groups (R-OM) that undergo hydrolysis–condensation reactions, resulting in enhanced stability [3]. Early examples of Class II hybrids include peptide-based macromolecules such as metallo-proteins [16] and synthetic water-soluble polymers, which are used for applications such as batteries [17], filtration systems [18], and the development of hydrophilic and hydrophobic materials [3].

This review highlights synthetic polymer–inorganic Class II hybrids, emphasizing the importance of strong chemical interactions, particularly coordination bonds, in defining the properties and applications of these materials with an emphasis on non-aqueous systems. Polymers with functional groups containing lone electron pairs, such as nitrogen, hydroxyl, or sulfur, can form coordination bonds with metal ions. Nitrogen-containing polymers, including polyamides, poly(ethylenimine) (PEI), and poly(vinylpyridine) (PVP), have been widely used for metal ion adsorption [19,20,21], catalyst resins [22,23,24], and biosensors [25,26]. The organic polymer component not only imparts structural support but also enhances the material’s mechanical properties and processability. For example, longer linkers provide greater flexibility, while shorter linkers create more mechanically robust frameworks, making them ideal for applications in gas separation and catalysis [27,28,29]. Functional groups on these linkers, such as carboxylates, amines, or hydroxyl groups, influence metal coordination, affecting stability, reactivity, and properties like conductivity and catalytic efficiency [30,31]. Additionally, the solubility and polarity of organic linkers are crucial for synthesis, as the homogeneity of the coordination complexes impacts the morphology and growth of the material [5].

The modularity between organic polymers and inorganic components in OIHs enables the design of materials that are highly adaptable for various applications. For example, by incorporating polymer coatings, these hybrids achieve enhanced solubility and colloidal stability, facilitating cost-effective processing [5,32]. These materials are particularly valuable for applications requiring long-term stability, such as magnetic nanoparticles for MRI contrast enhancement or ceramic nanocontainers used in drug delivery systems [32], nanopatterning [33,34,35], and microelectronic applications [36,37].

## 2. Methods of Synthesizing Coordinated Polymer–Inorganic Hybrids

The complexation routes for polymer–inorganic hybrids can pose significant challenges due to their low mixing entropy compared to small-molecule hybrids [38]. Furthermore, the poorly ordered macromolecular structure typically found in polymers could disrupt and alter the coordination geometry [38]. Therefore, the synthetic routes to polymer–inorganic hybrids need to be carefully curated to obtain specific products, such as when selecting precursors [39], reaction conditions [38,40], and necessary pre- [41,42] and post-processing techniques [43]. A comprehensive review of such routes is essential to understand the complexities of polymer–inorganic hybrids. In the synthesis of polymer–inorganic hybrid materials, various methods are employed, which can be broadly categorized into two main routes: homogeneous and heterogeneous phases. The homogeneous phase generally involves combining polymers and inorganic components directly in a solution. However, this method is exclusive to water-soluble complexes. On the other hand, the heterogeneous phase includes infiltration techniques such as vapor phase infiltration (VPI) and liquid phase infiltration (LPI) as post-process methods, providing more selective methods that extend beyond the limitations of hydrophilic polymers.

### 2.1. Homogeneous Synthesis

The synthesis of polymer–metal complexes, which forms the basis for creating organic–inorganic hybrids (OIHs), revolves around the coordination chemistry between metal ions and functional groups embedded within polymer chains. Typically, polymers equipped with functional groups such as pyridine, amine, or carboxylate groups are used because of their ability to coordinate strongly with metal ions. During synthesis, the polymer is dissolved in a compatible solvent, and a solution of the metal precursor is gradually introduced. The metal ions diffuse into the polymer matrix, interacting and binding with the available functional groups through coordination bonds. This method encompasses the early studies on polymer hybrids, and a more comprehensive review can be found elsewhere [44,45]. Nonetheless, examples of the most common polymers and metal precursors used to create these hybrids are provided in Section 3.

### 2.2. General Synthetic Route for Supramolecular Coordination Complexes (SCCs)

Supramolecular chemistry, which focuses on non-covalent interactions such as hydrogen bonding, metal coordination, and π-π stacking, plays a significant role in the development of organic–inorganic hybrids (OIHs). The field of SCC-based metallosupramolecular polymers has expanded rapidly in recent years, yielding exciting results and significant progress. These SCCs are characterized by their ability to form complex structures through self-assembly, which, when combined with polymers, has led to the development of two novel types of metallosupramolecular polymers [46]: metallacycle/metallacage-cored star polymers (MSPs) and metallacycle/metallacage-crosslinked polymer networks (MPNs) [47,48,49,50]. More details about supramolecular polymer chemistry are discussed in the last section of this review.

#### 2.2.1. Synthesizing Metallacycle/Metallacage-Cored Star Polymers (MSPs)

**Figure 1:**
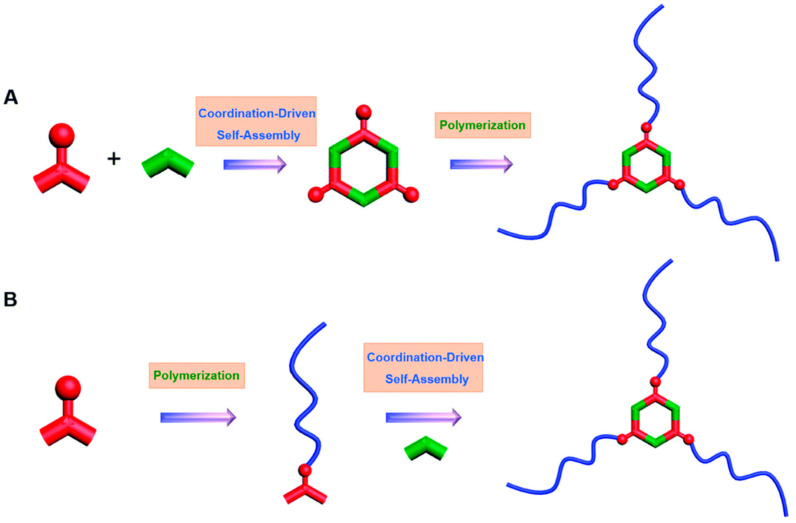
Synthetic approaches for the MSPs. (**A**) Post-assembly polymerization approach: metallacycles or metallacages are first formed via coordination-driven self-assembly, followed by polymerization of chains from the core. (**B**) Post-polymerization assembly approach: pre-synthesized macroligands with polymer chains and coordination sites undergo coordination-driven self-assembly to form MSPs. Red represents the metal ion, green represents the organic linker and blue represents the polymer. Adapted with permission from the Royal Society of Chemistry [52].

Metallacycle/metallacage-cored star polymers (MSPs) are branched polymers characterized by several linear chains connected to a central core, offering properties like compact structures, lower internal viscosity, and high arm density [51]. These characteristics make MSPs highly useful in applications such as drug delivery, thermoplastics, and nanoelectronics. Two synthetic approaches for MSPs are widely used: post-assembly polymerization and post-polymerization assembly. As shown in Figure 1A, in post-assembly polymerization (A), metallacycles or metallacages are first constructed through coordination-driven self-assembly, followed by the polymerization of multiple chains from these cores. In the post-polymerization assembly approach (B) (Figure 1B), macroligands with polymer chains and coordination sites are first synthesized, and then coordination-driven self-assembly forms the final MSPs [52].

#### 2.2.2. Synthesizing Metallacycle/Metallacage-Crosslinked Polymer Networks (MPNs)

**Figure 2:**
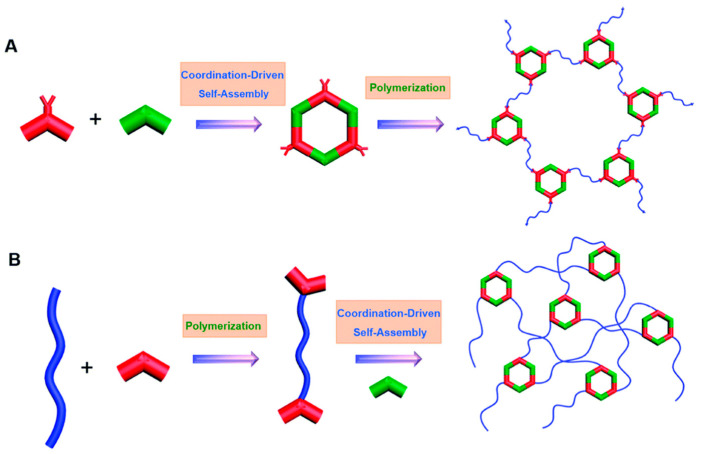
Synthetic approaches for the MPNs. (**A**) Post-assembly polymerization approach: metallacycles or metallacages with multiple reactive sites are first formed through coordination-driven self-assembly. These structures then undergo crosslinking at their reactive sites to create polymer networks with metallacycles/metallacages acting as crosslinking nodes. (**B**) Post-polymerization assembly approach: polymer chains with coordination sites are synthesized first, followed by coordination-driven self-assembly to form crosslinked MPNs. Red represents the metal ion, green represents the organic linker and blue represents the polymer. Adapted with permission from the Royal Society of Chemistry [52].

Similar to the synthesis of MSPs, two approaches, post-assembly polymerization and post-polymerization assembly, are employed for constructing MPNs. In the post-assembly polymerization method (as shown in Figure 2A), metallacycles or metallacages with multiple reaction sites are first created through coordination-driven self-assembly. These structures then undergo crosslinking at their reaction sites, forming the final polymer networks with metallacycles or metallacages acting as linkers. In the post-polymerization assembly method (Figure 2B), polymer chains with coordination sites are synthesized first, followed by coordination-driven self-assembly, to form MPNs [52]. The varied structures in MPNs present unique topologies and serve as valuable platforms for studying polymer networks and their physics [53]. Additionally, due to the well-defined pores or cavities in metallacycles and metallacages, MPNs are excellent candidates for applications such as gas separation, molecular sieving, and controlled release. These networks also provide a promising solution for improving the processability of widely used MOF materials, offering significant potential for practical applications [54].

### 2.3. Synthesizing Polymer–Inorganic Hybrids Through Vapor Phase Infiltration (VPI) and Liquid Phase Infiltration (LPI)

Hydrophobic and hydrophilic polymers can undergo infiltration, where inorganic precursors can be selectively introduced into specific domains of the polymer melt. Based on our findings from the available literature, the most common method for post-modification of coordination-based polymer–inorganic hybrids involves vapor phase infiltration (VPI) and liquid phase infiltration (LPI). The following section will explore these techniques in detail, discussing their processes and how they facilitate the formation of coordinated hybrid materials.

#### 2.3.1. Vapor Phase Infiltration (VPI)

**Figure 3:**
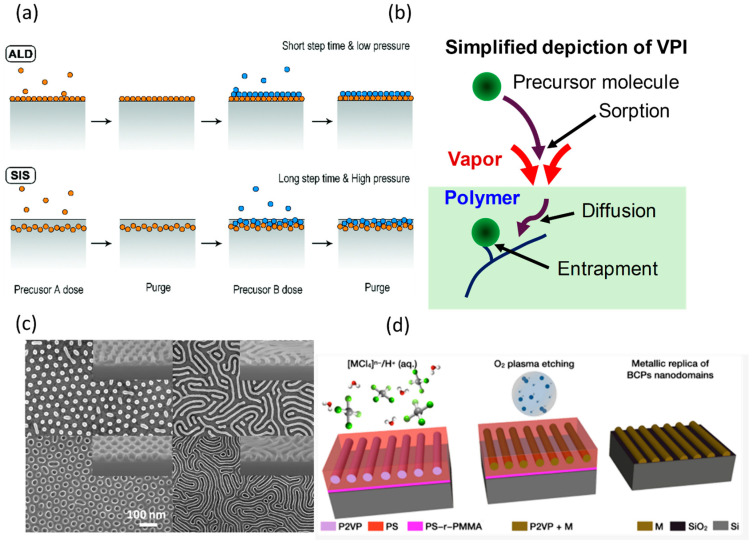
(**a**) Schematic of atomic layer deposition (ALD) and sequential infiltration synthesis (SIS) processes. ALD provides conformal surface coatings layer by layer, while SIS enables in-depth infiltration of precursors into polymer matrices [65]. (**b**) Schematic of vapor phase infiltration (VPI) process [68]. (**c**) Illustration of block copolymer (BCP) templating using annealing techniques as a pre-processing step for vapor phase infiltration (VPI). The self-assembled BCP thin film is annealed to achieve ordered domain structures, which serve as templates for selective infiltration and subsequent material synthesis [79]. (**d**) Schematics of liquid phase infiltration of BCPs: PS-b-P2VP cylinders are immersed in an acidic metal salt solution, where anionic metal complexes bind to protonated P2VP. O_2_ plasma then removes the polymer, reducing metal salts to nanostructures, replicating the BCP morphology [83]. Adapted with permission from the Royal Society of Chemistry [65,68] and American Chemical Society [79].

In vapor phase infiltration, a gas phase inorganic precursor infiltrates directly into the polymer melt host to form a polymer–inorganic hybrid [55]. VPI originates from atomic layer deposition (ALD); however, here, the precursor is allowed to “soak” for a prolonged period to allow infiltration into the polymer. The conversion of the hybrid into an oxide is then conducted by introducing an oxidizing agent, such as water, in a process known as sequential infiltration synthesis (SIS) [56]. In some literature sources, similar processes are introduced as sequential vapor infiltration (SVI), and multiple vapor infiltration (MPI) [57]. The fundamental idea of VPI is chemisorbing a precursor inside a polymer template in a facilitating environment, typically consisting of water vapor [58], ozone [59], or hydrogen peroxide vapor [60]. A few distinct differences between ALD and VPI usually extend to the requirements of the substrate: polar functional groups, relatively long deposition cycles, a high deposition rate, a nano to micro scale growth rate, the choice of the precursor, and the requirements of post-processing steps [57,61,62]. VPI is commonly used to enhance the mechanical properties of polymer matrices and etching efficiency [39,63,64]. The ALD and SIS processes are shown in Figure 3a [65].

The infiltration is carried out in three main steps (Figure 3b). First, the polymer template will be exposed to the precursor gas molecules, and sorption of gas molecules into polymers occurs on the surface. This is followed by diffusion of the gas molecules into the polymer matrix, and the concentration of the precursor determines the fraction of material transformed. Precursor–polymer solubility enhances infiltration, but this is not a requirement because VPI is performed after cycles of ultra-high vacuum, outgassing the polymer melt. This increases the free volume in the polymer matrix, permitting the diffusion of gas molecules despite limited solubility. This is a complex physiochemical process that is often simplified with classical continuum thermodynamics. In the case of pure dissolution, Henry’s law is used:

(1) C=S.p.

Here, *C*, the concentration of a penetrant molecule in a polymer, depends upon the partial pressure (*p*) of the penetrant species and its solubility coefficient (*S*) [66]. This solubility coefficient scales exponentially with temperature (*T*) according to the Van ’t Hoff equation [67]:

(2) $$S=S_{o}\exp\left[-\frac{\Delta H_{s}}{kT}\right]$$

where *S_o_* is a scaling constant, *k* is Boltzmann’s constant, and Δ*H_s_* is the partial molar enthalpy of sorption. While these equations do not provide an atomic-scale depiction of the dissolution phenomenology, they are useful for experimentally evaluating sorption processes [68].

This diffusion process is closely related to principles established in gas transport studies, where four primary categories of transport mechanisms are recognized: viscous flow, Knudsen diffusion, molecular sieving, and solution-diffusion [69,70]. To successfully complete the vapor phase infiltration (VPI) process, precursor molecules must be trapped within the polymer matrix. This entrapment typically occurs through chemical reactions between the precursor and functional groups in the polymer. However, other methods, such as reactions with a secondary precursor or physical trapping, can also be effective. The optimal scenario for VPI involves a tradeoff between diffusion and chemical bonding of the precursor molecules for complete infiltration into the polymer. Another strategy to achieve this moderate reactivity is by using polymer blends or copolymers, which combine highly reactive and non-reactive monomers, providing better control over the precursor’s diffusion and eventual entrapment in the polymer matrix [68].

The choices of primary precursors and secondary precursors are critical in VPI. The polymer must have polar sites where the precursor can be chemisorbed [71]. Furthermore, the operating temperature must be below that of the glass transition (Tg) of the selected polymer. This puts a constraint on the primary precursor, as it should be able to maintain very high vapor pressure below the respective Tg values [72]. These temperatures typically vary between 100 and 150 °C.

One of the common precursors used in VPI is trimethylaluminum (TMA), to synthesize aluminum oxide structures [58,73]. Metal halides such as TiCl_4_, MoCl_5_, SnCl_4_, alkoxides such as TIP, and metal alkyls like diethylzinc (DEZ) are also used in addition to TMA [55]. Polar polymers that can host such metal precursors range from biopolymers such as spider silk [63], egg collagen [64], polysaccharides [74], and cellulose [39], synthetic polymers such as Kevlar [75], epoxy-based photoresists [76], polyurethanes [77], polyaniline [78], P3HT, PMMA, PAA [38], and poly(vinylpyrrolidone), and block copolymers (BCPs) such as PS-b-PMMA [79] and PS-b-P4VP [43].

A major advantage in VPI is the area selectivity of the precursor due to chemical selectivity. Block copolymers (BCPs) are a common candidate for area-selective VPI and have been utilized for making 3D porous materials due to their ability to assemble into hierarchical structures [80,81,82]. Infiltrating such structures with inorganic precursors makes inorganic patterns and porous structures obtainable (Figure 3c). One of the limitations of VPI lies in its reliance on the polymer template’s uniformity. Unlike ALD, defects in the polymer may transfer into the final complex. As mentioned above, shrinkage of the porous structures could also be an issue for the quality of the final product. Some polymers may not have high infiltration dynamics, and additional pores are introduced by swelling [43]. These are prone to shrinkage in final processing due to mechanical instability. However, the major limiting factor is the Tg of the polymer materials. Due to the Tg ranging between 100 and 150 C for most polymers, the inorganic precursors must have high vapor pressure around the same temperature regime. This limits the library of inorganic precursors that can be used to infiltrate polymers using VPI [55].

#### 2.3.2. Liquid Phase Infiltration (LPI)

LPI is analogous to VPI, except a liquid phase inorganic precursor is used during infiltration. LPI involves the selective binding of a metal salt precursor to reactive functional groups within the polymer matrix, often through complexation or electrostatic interactions [83]. Similarly, it can be applied for area-selective infiltration of BCPs where one of the constituent blocks carries functional groups that facilitate this selective binding to create nanostructured inorganics (typically metals or metal oxides) that replicate the morphology of the polymer template [84] (Figure 3d). For BCPs, the metal precursor can be selectively incorporated either before the self-assembly process by mixing the BCP with the metal salt solution [85] or after self-assembly by soaking the formed nanostructures into the precursor-containing solution [34]. Alternatively, a metal salt solution can be directly spin-coated onto the polymer film [86].

LPI is sensitive to the pH value of the precursor solution due to the Lewis acid–base nature of the reactions. Often, an adjustment is made using hydrochloric acid [38], sodium hydroxide [87], or a buffer solution [88]. The pH alters the reaction time and temperature depending on the polymer and the inorganic precursor used [40,87,88]. Furthermore, LPI products can be obtained as a suspension, which requires extraction steps such as separation and freeze-drying [38,87]. Poly (acrylic acid) [38,87] and polyethyleneimine [88,89,90] are two common polymers used in LPI, and they are often paired with cyanate precursor.

While LPI can be a good candidate for mitigating vapor pressure limitations, enabling a greater diversity of inorganic precursors, a significant limitation of this method is the slower kinetics (reactions may require 24–72 h to complete [87,89,90]) compared to VPI, in which they are completed within minutes [91].

## 3. Polymer–Inorganic Coordinated Hybrids: Complexation-Induced Properties

Polymers with functional groups such as pyridine, imine, amine, and carboxylic acid are commonly employed due to their strong ability to coordinate with metal ions, enabling the formation of hybrid materials. The polymer–inorganic pair can be selected to modify thermal stability and electrical conductivity, and it imparts unique photophysical properties. They can also be designed for heterogeneous catalysts for chemical and biomedical manufacturing.

### 3.1. Pyridine-Based Polymers

Poly(4-vinylpyridine) (P4VP) and poly(2-vinylpyridine) (P2VP) are the two most studied polymers in coordination chemistry due to the nitrogen lone pair in their pyridine ring, which can form stable complexes with a variety of transition metals. The nitrogen atom acts as an electron donor, creating strong bonds with metal centers, resulting in new properties such as enhanced thermal stability and catalytic activity [92]. However, there are relatively fewer reports on P2VP coordinating with transition metals, likely due to its increased steric hindrance compared to P4VP. The nitrogen atom in P2VP is less accessible for coordination, making it less favorable for forming metal complexes than P4VP.

These complexes are particularly known for their applications in catalysis [93], environmental remediation [94,95], and sensing [96,97]. The structure of P4VP allows for easy coordination with metals such as copper, ruthenium, chromium, iron, zinc, aluminum, cobalt, nickel, and rare earth metals, making it a popular choice for creating hybrid materials used in diverse fields [96,98,99].

#### 3.1.1. Thermal Stability of Coordination Complexes of PVPs with Metal Ions

**Figure 4:**
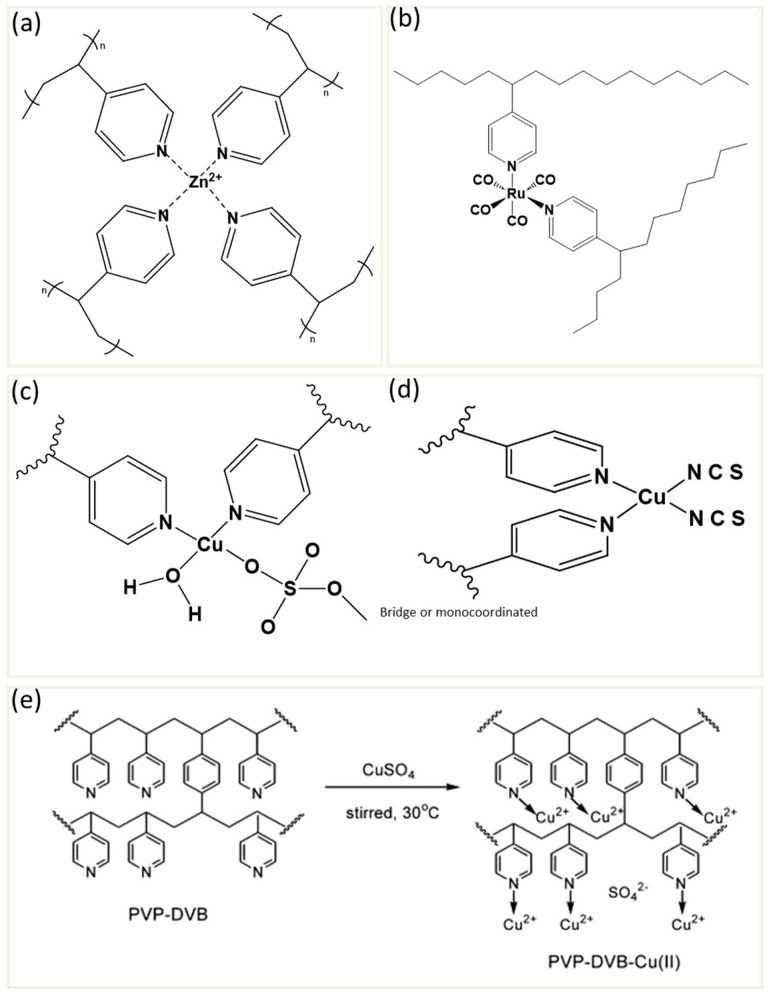
(**a**) Possible tetrahedral coordination complexation of ZnCl_2_ with P4VP [102]. (**b**) A ruthenium bridge between pyridine ligands on two different polymer chains illustrates the concept of coordination crosslinking [96]. (**c**) Schematic representation of P4VP–metal complexes: sulfate anions coordinated with Cu(II) in monocoordinated and bridged forms. (**d**) Thiocyanate coordinating through nitrogen, showing structural stabilization within the polymer matrix [93]. (**e**) Preparation of the PVP–DVB–Cu(II) complex [103]. Adapted with permission from Elsevier [93,96,103].

Previous research has demonstrated that thermophysical properties are modified synergistically when polymeric ligands occupy sites in the coordination sphere of the transition metal [100,101]. Many studies investigate the thermal stability of the compounds formed from P4VP and P4VP grafted with other polymers coordinated to transition metal salts. Zander et al. [102] investigated the impact of different metal salts, such as zinc (Zn^2+^), copper (Cu^2+^), and magnesium (Mg^2+^), on the glass transition temperature, Tg, of P4VP. The findings reveal that the type and geometry of the metal ion’s coordination with the pyridine rings of P4VP play a significant role in determining the extent of Tg enhancement. Among the tested metals, Zn^2+^ produces the most significant increase in Tg, achieving a maximum 70 °C rise at a loading of 0.25 molar equivalents, while other metals produce varied effects based on their unique coordination properties. Zn^2+^ forms a tetrahedral coordination complex (Figure 4a) with P4VP, which has a notable effect on the polymer’s Tg. This tetrahedral coordination creates a compact and highly crosslinked structure that restricts the mobility of polymer chains, resulting in the largest increase in Tg observed in the study. Cu^2+^ increases the Tg of P4VP, though less effectively than Zn^2+^ due to the formation of square planar coordination, which results in a less compact crosslinking structure. This geometry increases the distance between polymer chains, creating a lower level of rigidity and a smaller Tg enhancement compared to Zn^2+^. On the other hand, Mg^2+^ does not raise the Tg significantly, likely due to its smaller ionic radius and distinct coordination preferences that do not promote the same degree of crosslinking as Zn^2+^. Monovalent ions like Na^+^ and K^+^ have a lesser impact on the Tg, as they form primarily ionic interactions with P4VP rather than strong coordination bonds. These ions cannot create a crosslinked network within the polymer and thus show minimal Tg enhancement.

McCurdie et al. [96]. examined the effect of coordination between P4VP and dichlorotricarbonylruthenium(II) (RuCl_2_(CO)_3_)_2_. The Ru-based P4VP complex exhibited an enhancement of Tg by up to 50 °C [96]. This is driven by a few key mechanisms centered around the unique coordination properties of the Ru ion. Primarily, ruthenium’s ability to form strong coordination crosslinks with the nitrogen atoms on the pyridine rings in P4VP significantly increases the polymer’s thermal stability. Each Ru ion has the capacity to bridge two pyridine groups from different polymer chains, creating a network of coordination crosslinks. These crosslinks act as physical restrictions within the polymer matrix, reducing the mobility of the chains and thereby raising the Tg. This restriction of movement at a molecular level forces the system to maintain its solid-like structure until higher temperatures are reached, enhancing the Tg. Additionally, the electronic configuration of the Ru ion plays a crucial role in this effect. Here, ruthenium is in a d⁶ octahedral configuration, which is particularly stable due to ligand field stabilization energy. This stabilization energy arises from the strong interaction between the d-electrons of Ru and the surrounding ligands. An important aspect of this coordination involves π-backbonding, where electron density from Ru’s filled d-orbitals is donated back into the π* antibonding orbitals of the carbonyl ligands. This π-backbonding effect stabilizes the coordination complex, lowering the energy of the t_2_g molecular orbitals and enhancing ligand field splitting (Figure 4b).

In addition to polymer–metal interactions, it has been shown that the coordination environment of the complex could impact thermal degradation. Santana et al. [93] demonstrated that the degradation temperatures of the P4VP-Cu(II) complexes are generally lower than that of pure P4VP using thermogravimetric analysis (TGA). This reduction is likely due to alterations in the electronic density of the pyridine ring upon complexation, which reduces resonance-induced stabilization. The shift in the CN stretching band to higher wavenumbers, as observed in infrared spectroscopy, confirms these electronic density changes. Furthermore, coordination with Cu(II) ions, varying the anions, such as copper sulfate, chloride, and thiocyanate, significantly alters the degradation temperature of P4VP. For instance, sulfate complexes showed higher thermal stability compared to chloride and thiocyanate complexes. This is because the Cu(II)–sulfate complex (Figure 4c) coordinates with the nitrogen atoms of the pyridine rings in P4VP while also interacting with the sulfate ion in a monocoordinated or bridging configuration. This structure results in a symmetry change from the typical tetrahedral (Td) configuration of free sulfate ions to a lower symmetry, either C_2_v or C_3_v, due to coordination. This monocoordination or bridging arrangement stabilizes the complex by reinforcing the polymer’s crosslinked network, enhancing rigidity and limiting chain mobility, contributing to higher thermal stability. In contrast, the Cu(II)–thiocyanate complex (Figure 4d) does not form a bridge, leading to a less crosslinked structure compared to the sulfate complex.

Similarly, Wu et al. [103] showed that the degradation process of poly(4-vinylpyridine-*co*-divinylbenzene) block copolymer (PVP-b-DVB) begins at a higher temperature compared to the Cu(II)-complexed version, indicating that Cu(II) ions cause an initial reduction in thermal stability. This effect is thought to result from electronic density changes around the Cu(II) coordination sites, which weaken certain bonds in the polymer and lead to earlier degradation. The PVP-b-DVB copolymer primarily undergoes a two-stage degradation process, whereas the PVP-DVB-Cu(II) complex exhibits a three-stage degradation under a nitrogen atmosphere. This additional degradation stage, observed specifically in the Cu(II)-complexed samples, is associated with the breakdown of sulfate groups and Cu(II)–pyridine coordination bonds. Unlike thermal degradation, the Tg increased concomitantly with greater concentrations of Cu(II) ions, and this is attributed to the formation of crosslinks within the polymer matrix. The preparation of the complex and coordination of Cu(II) ions with pyridine groups are shown in Figure 4e.

#### 3.1.2. Electrical Conductivity of Coordination Complexes of PVPs with Metal Ions

Complexation of P4VP with metal ions is known to significantly enhance electrical conductivity [104,105]. The overlap of the frontier molecular orbitals of the metal atoms with the neutral basic pyridine ligands in the polymer allows for network formation and enhanced interchain electron transfer [106]. Rodriguez et al. [107] explored the enhancement of ionic conductivity in poly(2-vinyl pyridine) (P2VP) and P4VP upon complexation with copper iodide (CuI). This increase in conductivity, especially pronounced in P2VP/CuI complexes (from 4.6 × 10^−15^ to 4.2 × 10^−5^ S/cm), is likely a result of both cation and anion transport, considering that P2VP has sterically hindered Cu-N sites. The effect of ion mobility is further highlighted by temperature-dependent conductivity data, which revealed that the conductivity of the P2VP/CuI hybrids increases with temperature, particularly as the material nears its glass transition temperature (Tg). Saturating the CuI at 80% mol of P2VP reverted conductivity values back to those of pure P2VP, likely due to a lack of free volume.

Rafique et al. [104]. provide an in-depth examination of the electrical conductivity of P2VP complexes with different transition metals, copper (Cu), cobalt (Co), palladium (Pd), and platinum (Pt). While Cu complexation has a minimal effect on conductivity, Co increases conductivity by two orders of magnitude, Pd by six, and Pt by seven. The authors attribute this trend to several factors, including polarizability, ionization potential, metal ion size, intermetallic distances, and the rotational and vibrational dynamics of the metal ions. They highlight a clear correlation, where higher ionization potentials correspond to greater conductivity as the metal changes from Cu to Pt. The relatively lower conductivity of Cu- and Co-based complexes is attributed to ionic conductivity in agreement with their temperature dependence, wherein conductivity decreased with cooling. Surprisingly, Pd- and Pt-based P2VP complexes exhibit almost constant conductivity in the low-temperature region, which then declines at higher temperatures. Collectively, evidence suggests that charge transport could be governed by delocalized electrons through metal–metal orbital overlap. The close packing of Pd and Pt atoms supports electron delocalization along overlapping dz^2^ orbitals, forming a robust conduction pathway.

#### 3.1.3. Catalytic Activity of Coordination Complexes of PVPs with Metal Ions

**Figure 5:**
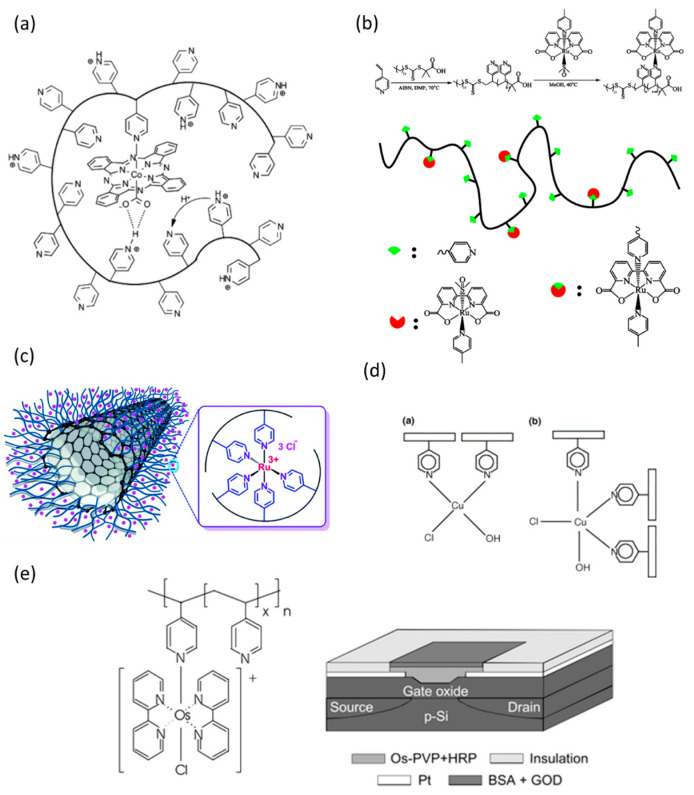
(**a**) An illustration of cobalt phthalocyanine (CoPc) encapsulated within a hydrophobic poly-4-vinylpyridine (P4VP) membrane highlighting the postulated primary-, secondary-, and outer-coordination sphere effects [109]. (**b**) Schematic synthesis of a P4VP–Ru polymer–metal complex by a combination of RAFT polymerization and a pyridine/DMSO exchange reaction [110]. (**c**) Proposed structure of Ru-PVP/CNT [111]. (**d**) Proposed Cu(II) structures in the fresh catalysts [112]. (**e**) Left: structure of the redox polymer osmium bipyridine PVP. Right: schematic cross section of the glucose-sensitive ^E^MOSFET [113]. Adapted with permission from the Royal Society of Chemistry [111,113] and Elsevier [112].

The complexes between P4VP and many kinds of transition metals have high catalytic activity for many reactions. The selective and efficient electrochemical reduction of CO_2_ to single products is crucial for solar fuel development. The catalytic activity of systems of cobalt phthalocyanine (CoPc) encapsulated within a P4VP membrane was investigated by Kamer and coworkers [108,109]. This system is designed to improve both the activity and selectivity of CoPc for CO_2_ reduction while suppressing the competitive hydrogen evolution reaction (HER). The goal is to selectively convert CO_2_ into useful products like carbon monoxide (CO) with high efficiency, which is crucial for renewable energy applications such as solar fuel development. The encapsulation of CoPc within P4VP significantly increases the catalytic activity for CO_2_ reduction compared to the parent CoPc complex. When CoPc is used alone, it shows modest activity and is not very selective, often generating a significant amount of H_2_ alongside CO. However, the CoPc-P4VP system demonstrates improved selectivity for CO_2_ reduction due to the unique coordination environment created by the P4VP polymer. The proposed mechanism for the enhanced catalytic activity of the CoPc-P4VP system involves multiple steps (Figure 5a). The authors describe three primary factors contributing to the system’s improved selectivity and activity for CO_2_ reduction over the hydrogen evolution reaction (HER). First, the axial coordination of the pyridyl groups from the P4VP to the Co(II) center of cobalt phthalocyanine (CoPc) increases the nucleophilicity of the metal center, facilitating stronger CO_2_ binding. This modification alters the rate-determining step of the reaction, shifting it from CO_2_ binding to a subsequent protonation event. Additionally, the polymer environment creates hydrogen bonding interactions in the secondary coordination sphere, which help stabilize the CO_2_ intermediates. The proton delivery to the active site is controlled by pyridine residues in the P4VP, which act as proton relays. This proton relay system selectively facilitates CO_2_ reduction by providing a slower, more controlled proton transport, which reduces proton availability for completing the HER. The encapsulated CoPc-P4VP complex, therefore, shows significantly higher selectivity for the CO_2_ to CO conversion, with much less H_2_ production compared to non-encapsulated CoPc.

Ruthenium complexes are among the most interesting coordination complexes, and they have attracted great attention over the past decades due to their appealing biological, catalytic, electronic, and optical properties. Ruthenium(II)-P4VP complex has been applied as a highly efficient water oxidation catalyst [110], taking advantage of the polymer’s flexible and charged environment. This system demonstrates impressive catalytic performance, with high turnover numbers (TONs) ranging from 1200 to 1700, making it more efficient than small-molecule analogs like [Ru(bda)(pic)_2_]. The coordination between P4VP and Ru in this system allows for a unique single-site water nucleophilic attack (WNA) mechanism, which differs from the commonly observed interaction between two metal oxide units (I2M) in other ruthenium-based catalysts. The P4VP polymer matrix plays a crucial role by preventing the dimerization of the Ru centers due to its multi-charge properties and slow diffusion characteristics, which enforce electrostatic repulsion and maintain the mononuclear catalytic pathway. Schematic synthesis of P4VP–Ru polymer–metal complex is shown in Figure 5b.

Ru-PVP complexes can be further enhanced by immobilization on carbon nanotubes for biomass conversion [111]. The catalyst showed selective aerobic oxidation of 5-hydroxymethylfurfural (HMF) to 2,5-diformylfuran (DFF). The functionalization process begins with the covalent grafting of PVP onto carbon nanotubes, which provides a robust matrix for immobilizing Ru(III) ions. The nitrogen atoms in the pyridine rings of PVP coordinate with Ru(III), creating a stable and active catalytic site. The coordination between Ru(III) and PVP allows the catalyst to operate effectively in an aqueous medium with molecular oxygen (O_2_) as the oxidant. This approach avoids the need for harsh chemicals or additional oxidants, making the process more environmentally friendly. The catalytic system demonstrated outstanding performance in the selective oxidation of HMF to DFF, achieving a yield of 94% under optimal conditions (at 100 °C and a reaction time of 6 h). The Ru-PVP/CNT catalyst (Figure 5c) demonstrates not only high catalytic efficiency but also selectivity toward DFF, minimizing side reactions and unwanted byproducts.

Additionally, there are many reports highlighting copper–P4VP metal complexes with applications for catalysis. Pardey and coworkers [112] explore the catalytic application of coordination complexes formed between dichlorocopper(II) and P4VP in a divinylbenzene matrix. P4VP–Cu complexes demonstrate catalytic activity in two significant reactions: the water–gas shift reaction (WGSR) and the reduction of nitrobenzene. In the WGSR, the Cu/P4VP complex facilitates the conversion of carbon monoxide and water into hydrogen and carbon dioxide. The nitrogen functionalities in P4VP enhance copper’s stability and catalytic efficacy in this reaction, making it comparable to traditional copper catalysts. Furthermore, in the reduction of nitrobenzene, the Cu/P4VP catalyst selectively converts nitrobenzene to aniline and, to a minor extent, azobenzene. Both reactions benefit from the coordination of copper with P4VP, which supports the copper species’ stability and catalytic performance under different conditions. Figure 5d presents two proposed structures for the fresh Cu(II)/P4VP catalyst: one with two pyridine ligands coordinated to copper and another with three pyridine ligands. However, based on EPR and XPS results, the structure with three pyridine ligands coordinated to copper is more likely predominant.

PVP–metal catalyst complexes have found applications in biomedical fields as well. Dam et al. [113]. present a highly sensitive glucose sensor that utilizes an Electrolyte Metal–Oxide–Semiconductor Field-Effect Transistor (EMOSFET) to measure glucose concentrations through potentiometric methods (Figure 5e). The sensor’s design includes a gate structure with glucose oxidase (GOD) immobilized within a bovine serum albumin (BSA) matrix using glutaraldehyde, layered on top of an electroactive osmium bipyridine poly(vinylpyridine) (Os-PVP) polymer that contains horseradish peroxidase (HRP). This innovative multi-layer system serves as the gate material for the FET, allowing it to detect glucose by measuring the work function changes of the electroactive gate material, which is influenced by the redox reaction involving hydrogen peroxide generated during the enzymatic reaction between glucose and glucose oxidase. The operating principle of the sensor is based on the detection of hydrogen peroxide (H_2_O_2_) as a byproduct of the glucose–glucose oxidase reaction. The presence of H_2_O_2_ leads to the oxidation of the Os-PVP layer, mediated by HRP. This oxidation process alters the gate’s work function, which in turn changes the threshold voltage (VT) of the EMOSFET. By applying a “constant current potentiometry” method, the sensor achieves a sensitivity that surpasses the typical Nernstian response, allowing for precise detection even at low glucose concentrations. A key feature of the sensor is the ability to control its sensitivity and detection limit by adjusting the reducing current applied to the gate. This current prevents complete oxidation of the Os-PVP, enabling the sensor to maintain a narrow and sensitive range. Additionally, the concentration of glucose oxidase within the enzymatic layer can be altered to affect the threshold voltage response, optimizing the sensor’s performance for various glucose concentrations.

#### 3.1.4. Photophysical Properties of Coordination of P4VP with Metal Ions

**Figure 6:**
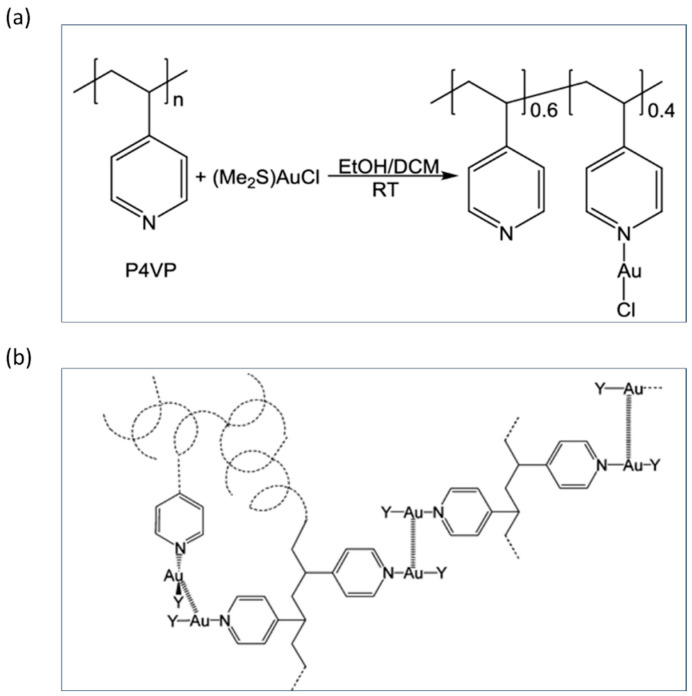
(**a**) Preparation of P4VP-Au (I) polymer complex: the synthesis involves the coordination of poly(4-vinylpyridine) (P4VP) with (Me_2_S)AuCl in a mixture of ethanol (EtOH) and dichloromethane (DCM) at room temperature [99]. (**b**) Suggested structural model showing intrachain and interchain aurophilic interactions in metallopolymers 1 and 2 [114]. Adapted with permission from the American Chemical Society [114].

Few studies have explored the impact of incorporating transition metals into polymers, which results in altered photophysical properties of the complex system. In a notable study, Hobbollahi et al. [99] explore the coordination behavior of poly(4-vinylpyridine) (P4VP) with two metal cations, Au(I) and Zn(II), forming luminescent metal-containing polymers. The synthesis of these metal-containing polymers involves reacting P4VP with gold and zinc precursors specifically, (Me_2_S)AuCl for the gold complex and ZnCl_2_ for the zinc complex (Figure 6a). Upon coordination, the polymers formed are insoluble in most common organic solvents, with the empirical formulas of (PVP)(AuCl)_0.4_ and (PVP)(ZnCl_2_)_0.7_. The Zn(II) complex was found to be highly crosslinked, while the Au(I) complex remained soluble in dimethyl sulfoxide (DMSO). The photophysical properties of these metal-containing polymers were analyzed, revealing that both P4VP-Au(I) and P4VP-Zn(II) complexes exhibit luminescence upon UV light excitation. The luminescence of the P4VP-Au(I) complex is primarily attributed to aurophilic interactions, where gold–gold (Au-Au) contacts contribute to the emission properties. On the other hand, the luminescence of the P4VP-Zn(II) complex is excitation wavelength-dependent, which is proposed to result from the formation of aggregated species with different excitation energies. These aggregates create a complex emission profile that varies with the wavelength of excitation.

In a similar study, Omary et al. [114]. investigate the synthesis, photophysical properties, and structural aspects of a novel class of gold-based metallopolymers. These metallopolymers are formed by coordinating P4VP with pentahalophenyl-gold(I) precursors, resulting in materials with intriguing phosphorescent properties and enhanced thermal and photonic stability. Two metallopolymers, [Au(C_6_F_5_)PVP] (1) and [Au(C_6_Cl_5_)PVP] (2), were synthesized by reacting P4VP with the respective gold(I) precursors. One of the most striking properties of these metallopolymers is their bright photoluminescence (PL) in the solid state. Temperature-dependent PL spectra reveal that both 1 and 2 exhibit tunable emission maxima based on the excitation wavelength, with luminescence colors ranging from blue to yellow, depending on the temperature. This thermochromic luminescence is highly sensitive to temperature, which indicates the presence of active triplet (T1) states within the metallopolymers. The paper similarly suggests that the emission properties and stability of these metallopolymers are due to aurophilic (Au(I)···Au(I)) interactions within the polymer matrix. These interactions are both intra- and interchain, forming a network that stabilizes the material’s luminescent state. A structural model, illustrated in Figure 6b, shows these interactions, where each Au(I) ion is influenced by neighboring Au(I) centers. This structural feature enables the metallopolymers to maintain phosphorescent properties across a range of temperatures and excitation conditions. The paper discusses the potential for these metallopolymers to be used in polymer light-emitting diodes (PLEDs), leveraging their high solid-state quantum yields, energy-rich triplet excitons, and fast phosphorescence lifetimes.

### 3.2. Therapeutics, Sensing, and Polyelectrolytes

Polyethyleneimine (PEI) is a common choice for forming complexes with metals due to its abundant free amino groups, which can coordinate with metal ions through electrostatic interactions or coordination bonds. PEI contains primary, secondary, and tertiary amines, with approximately 25% of the nitrogen atoms being primary, 50% secondary, and 25% tertiary. The study emphasizes that the binding of metal ions is mainly facilitated by these amine groups, with primary amines playing a particularly crucial role. When PEI is chemically modified by acetylation or alkylation, which blocks the primary amines, there is a significant decrease in the polymer’s ability to bind metal ions. The mechanism of metal ion complexation described by Kozuka and coworkers [115] involves a cooperative binding process. The binding of metal ions, especially Cu^2+^, induces a conformational change in the PEI structure, causing the polymer chains to swell or unfold. This unfolding increases the exposure of additional amine groups that were previously buried within the polymer matrix, allowing more metal ions to bind. This behavior is evident in the cooperative nature of the binding isotherms, where there is a steep increase in metal ion uptake as the free metal ion concentration rises, particularly pronounced for Cu^2+^.

PEI is commonly employed in a range of applications, including biomedical, molecular imaging [116], drug delivery [117,118], and wastewater treatment. Its strong metal-binding properties make it suitable for designing metal-based complexes, particularly for DNA binding in anticancer therapies. PEI is regarded as one of the most efficient polymers for transfection, both in vitro and in vivo. Its high density of amine groups contributes to its DNA-condensing ability and its interaction with cell membranes, facilitating uptake. However, a significant drawback of PEI is its high cationic charge density, which is associated with cytotoxicity. While lower-molecular-weight PEI is less toxic, it also demonstrates reduced transfection efficiency. This tradeoff has driven research efforts to modify PEI, such as incorporating biodegradable linkages, to improve its biocompatibility without sacrificing its effectiveness. These modifications aim to create safer PEI-based systems for clinical applications [119].

**Figure 7:**
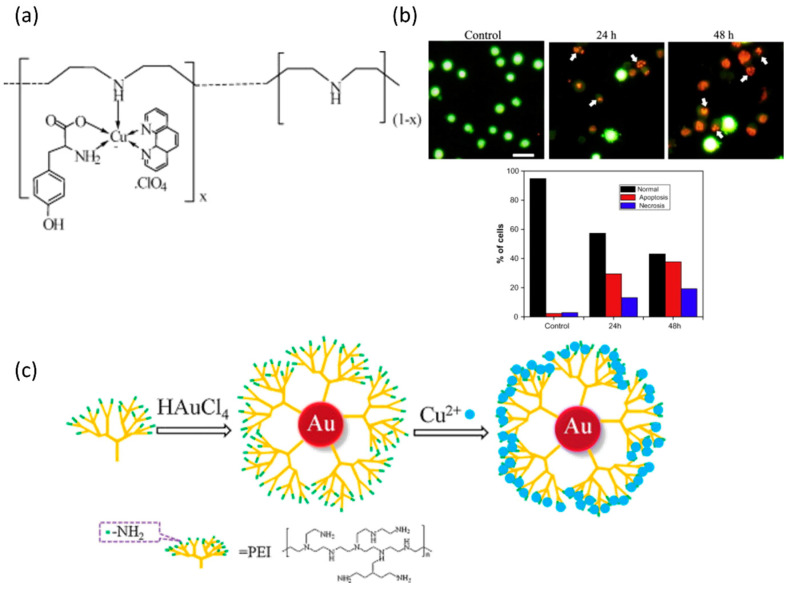
(**a**) Schematic representation of [Cu(phen)(L−tyr)BPEI]ClO_4_. (**b**) Morphological analysis of MCF−7 cancer cells stained with acridine orange and ethidium bromide after treatment with [Cu(phen)(L−tyr)BPEI]ClO_4_. The images show a time−dependent increase in apoptosis, indicated by chromatin condensation and cell shrinkage. Scale bar: 35 µm [120]. (**c**) Schematic illustration for fabrication of PEI/Au NPs for colorimetric detection of Cu^2+^ [121]. Adapted with permission from Elsevier [120,121].

Lakshmipraba et al. [120] synthesized water-soluble PEI–copper(II) complexes using phenanthroline and L-tyrosine as co-ligands (Figure 7a). Their study showed that the DNA-binding affinity of these complexes increased with the number of copper centers in the polymer. Thermal denaturation studies indicated that higher coordination degrees contribute to improved DNA stability, as the complexes stabilize the DNA double helix. The paper also describes significant cytotoxic activity of the most highly coordinated polymer–copper(II) complex against MCF-7 breast cancer cells, with apoptosis being the primary mode of cell death, confirmed through various assays that detect DNA fragmentation and apoptotic morphology. Figure 7b further supports these findings by showing AO/EB-stained photomicrographs of MCF-7 cells treated with the complexes, highlighting apoptotic changes such as chromatin condensation and membrane blebbing, alongside quantitative data indicating an increase in apoptotic cells over time.

PEI-Au hybrids have been developed for simple and sensitive colorimetric sensors for detecting copper ions (Cu^2+^) (Figure 7c) [121]. The team synthesized the PEI/Au NPs by employing polyethyleneimine (PEI) as a reducing and stabilizing agent without the need for additional nanoparticle seeds or stabilizers. The detection mechanism hinges on the specific interaction between Cu^2+^ ions and the amino groups present on the PEI, which results in a color change from wine red to purple, providing a visual indication of a copper ion presence. The binding of the Cu^2+^ ions with the PEI-capped nanoparticles caused a new absorption peak to emerge in the UV/Vis spectrum and changed the solution color, which was attributed to the formation of the Cu-PEI complex. To validate the sensor’s performance, the authors tested it on real water samples, including wastewater from a local steel works and river water. The PEI/Au NP sensor provided accurate Cu^2+^ concentration measurements, demonstrating its potential for practical applications in environmental monitoring.

Polyacrylic acid (PAA) chains offer numerous carboxylate groups, which are commonly used as polyelectrolytes. Qi et al. [38] observed how polyacrylic acid (PAA) interacts with lanthanide ions to form polymer–metal complexes, focusing on the impact of pH and the ionization behavior of PAA. The researchers found that the binding mechanism is highly dependent on the pH of the solution. At low pH levels, the carboxylate groups of PAA are not fully ionized, resulting in minimal interaction with lanthanide ions. As the pH increases, more carboxylate groups become deprotonated, enhancing their negative charge and ability to coordinate with the positively charged metal ions. This increased ionization facilitates stronger and more stable complex formation, which becomes visually apparent through the precipitation observed at higher pH values.

### 3.3. Nano-Templated Hybrids

Block copolymers (BCPs) are highly valued for high-resolution patterning due to their simplicity, and high throughput capabilities. These polymers naturally self-assemble into a range of well-ordered nanostructures, offering tunable resolutions from a few to several hundred nanometers due to the chemical immiscibility of their covalently bonded segmental chains [122,123]. High-resolution functional nanostructures can be efficiently produced by using these BCP microdomain masks in different templating processes. Since the two blocks in di-BCPs feature distinct chemistries, it becomes feasible to selectively introduce precursors, such as metal ions or molecules, into the self-assembled structure and create mesoscaled self-assembled hybrids. This targeted infiltration of BCP sites can be achieved through various methods such as vapor phase methods, like sequential infiltration synthesis (SIS), or through liquid phase precursors.

#### 3.3.1. Nanomaterials as Sacrificial Templates

**Figure 8:**
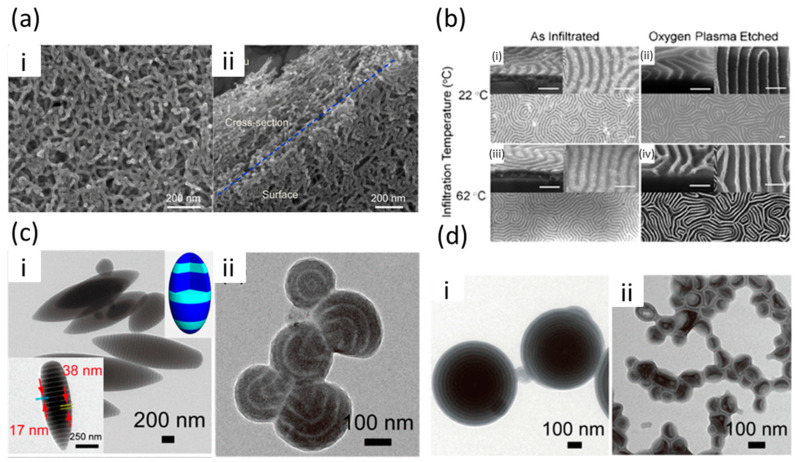
(**a**) (**i**,**ii**) SEM images of TiO_2_ nanowires with a random alignment templated from self-assembled PS-b-PMMA BCP film on a QCM crystal after 8 SIS cycles at 135 °C [125]. (**b**) (**i**,**ii**) Top-view and cross-sectional SEM images of as-infiltrated self-assembled PS-b-P2VP BCP thin films after Pt LPI for 75 s at 22 and 62 °C, respectively. (**iii**,**iv**) After oxygen-plasma ashing of the polymer matrix from the Pt-infiltrated PS-b-P2VP BCP thin films in (**i**,**iii**). All scale bars denote 100 nm [34]. (**c**) TEM images and the corresponding schematic illustrations (upper-right insets) of the PS_9.8k_-b-P4VP_10k_-Pb(II) particles obtained by solvent evaporation of emulsions containing PS_9.8k_-b-P4VP_10k_ in a chloroform phase (10 mg/mL) and Pb(II) ions with varied concentrations, (**i**) 0 M and (**ii**) 0.04 M, in PVA aqueous solution (3 mg/mL) [128]. (**d**) TEM images of the PS_9.8k_-b-P4VP_10k_-Fe(III) assemblies obtained by solvent evaporation of emulsions containing PS_9.8k_-b-P4VP_10k_ in the chloroform phase (10 mg/mL) and Fe(III) with varied concentrations, (**i**) 0.001 M and (**ii**) 0.1 M, in PVA aqueous solution (3 mg/mL) [128]. Adapted with permission from the American Chemical Society [34,125,128].

Using a sacrificial template offers an efficient and straightforward method for creating patterned nanomaterials through vapor phase infiltration (VPI). While various templates and processes are available, they all follow a similar procedure. Initially, a polymer template is infiltrated with an inorganic material through VPI. During this process, the precursors react with the organic template, forming a hybrid organic–inorganic material. Since mineralization takes place within the template, its morphology is preserved post-hybridization. Finally, calcination, reactive ion etching, or UV or ozone cleaning of the hybrid material produces an inorganic replica of the original template, often resulting in a porous structure [124]. An example of this approach involves the infiltration of TiO_2_ in PS-b-PMMA BCP films. Over multiple SIS cycles, the accumulation of TiO_2_ within the PMMA domains gradually reduced the permeability of the polymer, ultimately restricting further infiltration. Once the desired level of infiltration was achieved, the BCP template was removed by calcination, leaving a structured inorganic TiO_2_ framework that replicated the morphology of the original BCP nanodomains (Figure 8a) [125]. However, this step must also consider the template’s shrinkage and temperature sensitivity to preserve the porous structures [43].

As mentioned above, liquid phase infiltration has also been vastly used to synthesize BCP-templated hybrid films and directly patterned inorganic nanostructures [84,126,127]. Subramanian et al. [34] used polystyrene-block-poly(2-vinylpyridine) (PS-b-P2VP) BCP films with a self-assembled lamellar structure as templates. By soaking these films in a solution containing platinum ions (Na_2_PtCl_4_) at elevated temperatures, they achieved selective infiltration of Pt ions into the P2VP domains, exploiting the pyridine groups’ affinity for metal ions. The increase in temperature enhanced the infiltration rate and amount of Pt loading in the P2VP domains, producing a denser metal-loaded structure. After sufficient infiltration, the polymer matrix was removed by oxygen plasma ashing, leaving behind a patterned Pt nanowire structure that replicated the original morphology of the BCP (Figure 8b).

#### 3.3.2. Hybrid Induced Self-Assembled Nanomaterials

In recent years, the integration of inorganic components into BCP systems has opened new pathways for designing hybrid nanomaterials. Incorporating inorganic components into BCPs not only expands the range of achievable properties but also influences the morphology of the resulting hybrid system. In a study by Wu et al. [128], metal ions of Pb(II) and Fe(III) were incorporated into polystyrene-block-poly(4-vinylpyridine) (PS-b-P4VP). This study employed 3D confinement in emulsion droplets, where the PS-b-P4VP was confined within tiny droplets during solvent evaporation, leading to unique structural formations. The selective reaction of Pb(II) with 4VP units resulted in shrinking the P4VP domains through crosslinking interactions. As the concentration of Pb(II) ions increased, the morphology of the PS-b-P4VP particles transitioned from lamellar (layered) to bent lamellar, eventually forming spherical domains. This morphological shift resulted from the change in geometric packing of the P4VP domains due to Pb(II)-induced crosslinking, which altered the overall shape and internal structure of the assemblies (Figure 8c).

Fe(III) ions, in contrast, introduced a different type of transformation. Initially, at low Fe (III) concentrations, the P4VP chains formed a coordinated layer around the emulsion droplets, stabilizing an “onion-like” structure where the P4VP domains concentrated near the droplet surface. However, at higher concentrations, Fe(III) ions hydrolyzed, lowering the pH and causing protonation of the P4VP units. This protonation made P4VP more hydrophilic, which decreased interfacial tension and caused instabilities in the emulsion droplets, leading to the breakup of the larger droplets into nanoscale vesicles and other smaller structures (Figure 8d).

## 4. Polymer–Inorganic Hybrids as Linkers

**Figure 9:**
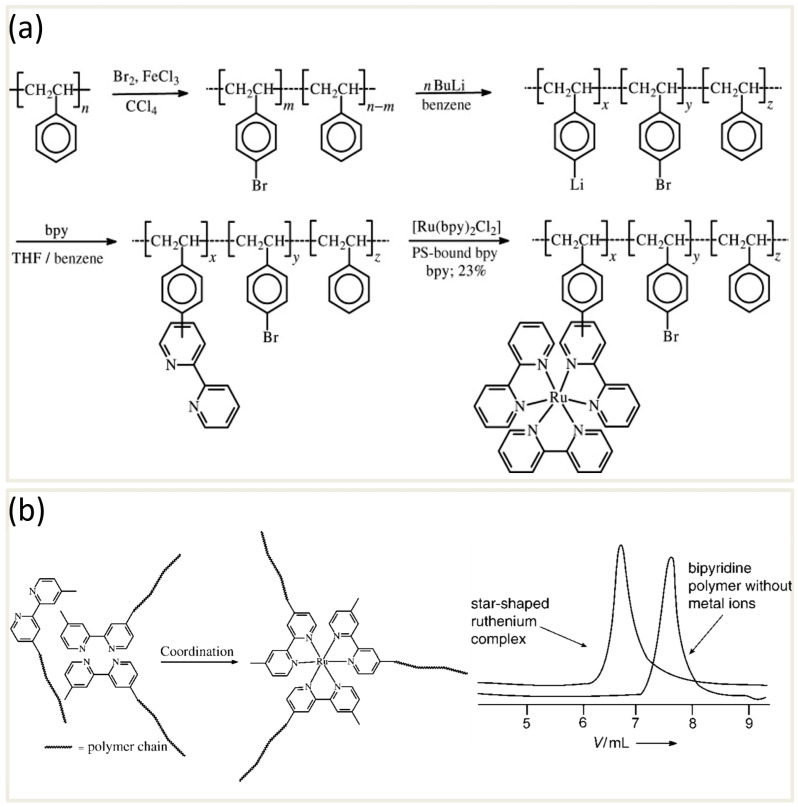
(**a**) Synthesis of the first polymers with pendant [Ru(bpy)_3_] complexes. This complex is synthesized via radical polymerization of a polystyrene backbone, followed by bromination, lithiation, and functionalization with 2,2′-bipyridine. (**b**) Left: preparation of star-shaped polymers by the addition of metal ions to monofunctionalized bipyridine polymers; right: GPC analysis showed a significant shift in the molecular weight after complexation. Adapted with permission from John Wiley and Sons [135].

Another well-defined category of organic–inorganic hybrids is metallosupramolecular complexes, which combine the functionalities of metal ions with organic ligands to form highly structured materials. The term “metallosupramolecular chemistry” was introduced by Constable in 1994 to describe assemblies where metals act as a type of “glue,” holding together organic molecules through metal–ligand coordination [129]. Over the past few decades, this field has experienced exponential growth due to its ability to produce functional, stimuli-responsive materials with diverse applications. Metallosupramolecular polymers (MSPs) are a specific subset within this broader field, formed by the self-assembly of macromolecular monomers equipped with metal-binding motifs [52]. These materials utilize non-covalent interactions, including metal–ligand complexation, hydrogen bonding [130,131], and host–guest interactions [131], which provide them with dynamic, adaptable properties. Because of the non-covalent nature of these interactions, MSPs are highly responsive to external stimuli, such as heat, light, chemicals, mechanical force, and electric or magnetic fields, allowing for temporary disassembly into monomeric units [132,133]. This dynamic responsiveness supports functions such as self-healing, reversible adhesion, recycling, and easy reprocessing, making MSPs attractive for sustainable material design [49,52,134]. A comprehensive review of metallosupramolecular polymers is reported by Schubert et al. [135], which covers numerous examples in the field, including pioneering work by Kaneko et al. [136]. In 1980, Kaneko and colleagues presented one of the first studies on polymers with covalently attached bipyridines, aiming to develop a heterogeneous photocatalyst for solar-driven water decomposition. They synthesized these polymers by radical polymerization of a polystyrene backbone, followed by bromination, lithiation, and subsequent modification with bipyridine at various molar ratios (Figure 9a). To introduce ruthenium (Ru) into the polymer, they coordinated the bipyridine-modified polymer with [Ru(bpy)_3_]^2+^. This coordination endowed the polymer with photocatalytic properties, enabling it to absorb light and drive redox reactions essential for water splitting.

One of the key features of MSPs is the metal–ligand interactions, which can vary significantly in strength depending on the specific metal and ligand involved. A notable class of ligands used in metallosupramolecular chemistry includes nitrogen-containing heterocyclic ligands, such as those with pyridine or imidazole units [129,134]. These ligands are versatile and widely employed due to their ability to form stable complexes through their multiple donor atoms and the angular orientations of the metal-binding domains. An interesting example of this class of MSPs involves polymers containing a single bipyridine unit in the backbone. When metal ions are introduced to this bipyridine moiety, star-shaped polymer structures are formed. Chujo et al. [137] synthesized such polymers by substituting bipyridines on only one side with poly(ethylene oxide) or poly(propylene oxide) chains (Figure 9b), beginning with 4,4′-dimethylbipyridine. By reacting with LDA and subsequently adding an α-alkyl ether-ω-tosylate polymer, they produced the desired structure, containing a single bipyridine unit. The addition of metal ions like Ni^2+^, Co^2+^, or Ru^3+^ resulted in star-shaped configurations, which were analyzed via UV/Vis spectroscopy and GPC. Notably, the Ru complexes exhibited stability under GPC shearing conditions, while the Ni^2+^ and Co^2+^ complexes dissociated.

The architecture of metallosupramolecular assemblies can be controlled through ligand design. For example, linear coordination polymers, one of the simplest metallosupramolecular structures, are formed by combining linear bridging ligands with metals that provide 180° linkages, such as silver(I), palladium(II), or iron(III). These polymers extend in only one dimension, but by altering the structure of the ligand, more complex architectures, such as zigzag coordination polymers, can be achieved [129]. This tunability highlights the power of metallosupramolecular chemistry in designing materials with desired structural and functional properties. One of the most appealing aspects of metallosupramolecular chemistry is its simplicity and efficiency compared to traditional synthetic methods. While organic synthesis often involves complex, stepwise procedures with limited yields, the formation of metallosupramolecular structures typically occurs quantitatively through spontaneous self-assembly. By simply mixing the appropriate metal and organic ligand building blocks, the desired product can often be obtained in a single step. This self-organization process is driven by molecular recognition, where the coordination geometry of the metal ions and the spatial arrangement of donor atoms in the ligands determine the final structure [129]. Thus, MSPs boast a combination of processability and functional properties, offering a powerful platform for developing advanced materials with applications in fields such as smart materials, catalysis, and drug delivery systems.

Metallosupramolecular polymers, in general, are classified into three main categories: metal–organic frameworks (MOFs), coordination polymers (CPs), and supramolecular coordination complexes (SCCs). Historically, the terminology surrounding coordination polymers (CPs) and metal–organic frameworks (MOFs) has often been used interchangeably, leading to some confusion. While they do share certain similarities, such as being composed of metal ions coordinated to organic ligands to form extended structures, they typically refer to small-molecule linkers, which are not the emphasis of this review. For the sake of accuracy, we will briefly discuss each and highlight the distinctions between them.

### 4.1. Coordination Polymers (CPs) and Metal–Organic Frameworks (MOFs)

**Figure 10:**
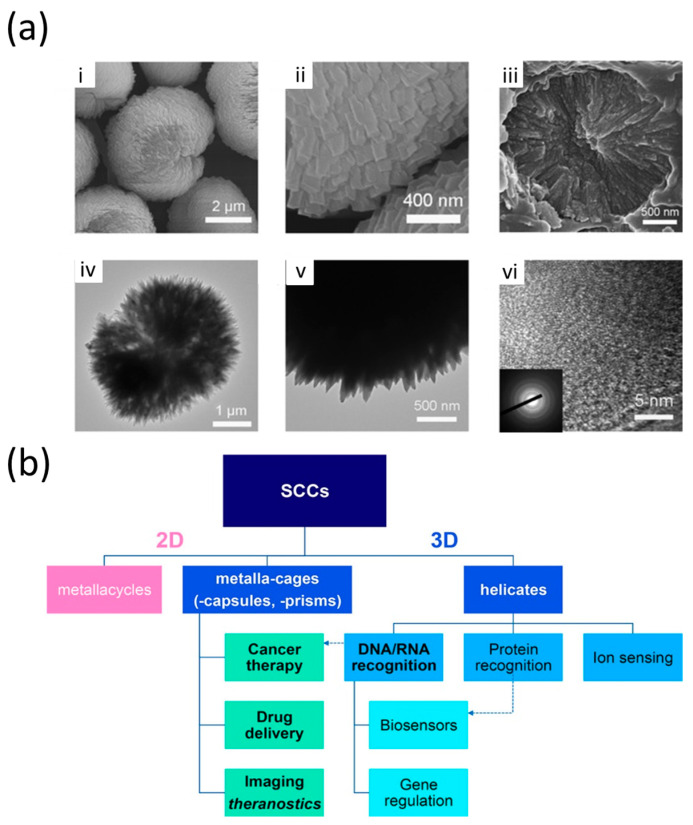
(**a**) (**i**) SEM image of Cys(Zn) microspheres obtained from the assembly of cystine (2 mm) and ZnCl_2_ (2 mm) at pH 8.0. (**ii**) Enlarged SEM image of the edge of a Cys(Zn) microsphere. (**iii**) SEM image of a section of a Cys(Zn) microsphere. (**iv**) TEM image of a Cys(Zn) microsphere. (**v**) Enlarged TEM image of the edge of a Cys(Zn) microsphere. (**vi**) High-resolution TEM image and SAED pattern (inset) of the nanorods [140]. (**b**) Schematic representation of the various types of SCCs and the scope of their biological applications. [46] Adapted with permission from John Wiley and Sons [139] and the American Chemical Society [46].

Coordination polymers are metal–ligand coordination compounds that form one-dimensional (1D) extended chains, two-dimensional (2D) sheets, or three-dimensional (3D) frameworks [138]. The dimensionality of CPs exhibits considerable variation, ranging from linear chains to intricate flexible networks. The use of flexible ligands with rotatable covalent bonds, involving sp³ hybrid atoms, like carbon, oxygen, or nitrogen, allows for a wide range of conformations during the self-assembly process. For example, a recent study showed that a zinc–cystine coordination polymer can self-assemble into a 3D structure resembling chloroplasts, combining light-harvesting and catalytic functions for sustainable fuel production (Figure 10a) [139]. CPs have also demonstrated promising applications in biomedicine due to their versatility and multifunctionality. These applications span drug delivery, bioimaging, and biosensing applications. Nanoscale coordination polymer particles (CPPs), for example, can encapsulate therapeutic agents, protecting drugs from degradation and minimizing side effects while also enabling targeted delivery to cancer cells [140].

On the other hand, MOFs represent a specific category of CPs, characterized by their three-dimensional (3D) structures that frequently display considerable porosity with tunable pore sizes [141]. These properties make MOFs ideal for applications like catalysis, where the increased surface area supports active catalytic substances, while the adjustable pores can selectively filter molecules for specific reactions. Furthermore, MOFs serve as effective delivery systems for a variety of therapeutic agents, including drugs, nucleic acids, proteins, and dyes. Their large surface area, tunable pore sizes, and biocompatibility allow for the efficient encapsulation of these cargos, making them highly versatile in biomedical applications. A detailed review by Jie Yang and Ying-Wei Yang provides an in-depth analysis of MOFs and their wide-ranging applications in the biomedical field, offering valuable insights into their synthesis, functionalization, and use in areas like drug delivery, bioimaging, and cancer therapy [142].

In theory, the length of organic linkers scales with the pore size of MOFs within a given network. Recently, the concept of supramolecular templating has emerged as an alternative approach for creating hierarchically ordered MOFs. These templates can be classified as hard, such as polystyrene, or soft, including micelles, surfactants, block co-oligomers, and others [143]. In both compounds, coordination chemistry serves as the foundation for both CPs and MOFs, where metal ions interact with organic ligands to create extended structures via coordination bonds [144,145,146].

### 4.2. Supramolecular Coordination Complexes (SCCs) Including Two-Dimensional (2D) Metallacycles and Three-Dimensional (3D) Metallacages

SCCs, on the other hand, are discrete molecular entities formed through the coordination-driven self-assembly of suitable metal centers and ligands containing multiple binding sites [46,147]. The combination of SCCs and polymers has resulted in hybrid materials with highly diverse and well-defined topological architectures, offering a platform for exploring their potential in various fields. The field of SCC-based metallosupramolecular polymers has expanded rapidly in recent years, yielding exciting results and significant progress. SCCs, including two-dimensional (2D) metallacycles and three-dimensional (3D) metallacages, have demonstrated wide-ranging applications in host–guest chemistry, sensing, catalysis, and drug delivery. These SCCs are characterized by their ability to form complex structures through self-assembly, which, when combined with polymers, has led to the development of two novel types of metallosupramolecular polymers [46]: metallacycle/metallacage-cored star polymers (MSPs) and metallacycle/metallacage-crosslinked polymer networks (MPNs) [47,48,49,50]. In these hybrid materials, SCCs function as either core structures or crosslinking units, enhancing both mechanical properties and responsiveness to external stimuli. The highly connected SCC cores or crosslinks impart these materials with unique mechanical properties and stimuli responsiveness, making them ideal candidates for smart materials that can adapt to changing environmental conditions. Moreover, the well-defined nanopores or nanocavities of SCCs, combined with the processability of polymers, make these hybrid materials promising for practical applications as porous, soft materials, particularly in molecular separations. As shown in Figure 10b, SCCs can be classified into two main structural categories: 2D metallacycles and 3D helicates. These structures have a wide range of therapeutic applications. For example, metallacages (which include capsules and prisms) are used in cancer therapy, drug delivery, and imaging theranostics. Additionally, SCCs play significant roles in DNA/RNA recognition, biosensors, protein recognition, gene regulation, and ion sensing. The unique properties of SCCs, such as their ability to form precise molecular architectures, make them ideal for targeted applications in biological systems [46].

## 5. Conclusions

In summary, this review highlights the synthesis, classification, and applications of metal-coordinated polymer–inorganic hybrids, focusing on materials that incorporate polymers through coordination with metal ions. We first examine key synthetic methods, including homogeneous and heterogeneous approaches. Homogeneous synthesis involves directly combining polymers and inorganic components in solution, making it particularly suitable for water-soluble polymers. In contrast, heterogeneous synthesis uses liquid and vapor phase infiltration as post-processing techniques, allowing for the selective incorporation of metal precursors into specific polymer domains. Furthermore, we classify these hybrids based on the functional properties they gain after complexation, focusing on enhanced electrical conductivity, thermal stability, catalytic activity, and tunable photophysical characteristics. An essential advantage of these hybrids is their templating capabilities: they can either create hybrid-induced self-assembled structures or act as frameworks where one component can be removed, leaving a stable scaffold of the remaining material.

Additionally, these hybrids serve as linkers, connecting polymer chains through metal coordination to form metallosupramolecular polymers. This group of hybrids includes three main categories: metal–organic frameworks (MOFs), coordination polymers (CPs), and supramolecular coordination complexes (SCCs), such as 2D metallacycles and 3D metallacages. These metallosupramolecular structures offer unique structural and functional properties that are highly advantageous for applications in sensing, catalysis, and drug delivery.

Looking ahead, future research in polymer–inorganic hybrids will likely focus on expanding the variety of polymer and metal pairings to create hybrids with increasingly tailored properties for specialized applications. An exciting direction involves exploring the coordination of Group IV semiconductors, such as silicon and germanium, with polymers and block copolymers. Given their tunable band gaps, these semiconductors could impart unique electronic and optical properties to hybrid complexes, making them highly valuable for optoelectronic applications and next-generation materials with programmable properties.
